# Supplementary material for: Mountain moves: Spatial interaction modelling of Bulgaria’s internal migration (1934-1992)
Source: PLoS One. 2026 Mar 2;21(3):e0341180. doi: 10.1371/journal.pone.0341180 (PMC12952610; doi:10.1371/journal.pone.0341180)
Supplement: Attachment — Submitted filename: Reviewrs report PONE-D-25-23423.pdf [file pone.0341180.s001.pdf]

Date: August 13, 2025

Dear Authors,

Thank you very much for conducting such an intriguing and timely study. While your manuscript "Mountain Moves: Spatial Interaction Modelling of Bulgaria's Internal Migration (1934-1992)" is highly commendable, I recommend addressing the following key comments to enhance its overall quality and clarity. To facilitate further review, please clearly highlight all revisions in the updated manuscript.

Abstract:

- Could you please include the existing migration situation in Bulgaria so that the reader of your manuscript can have a full understanding of the situation and the necessity of your study for development activities, including policy advice or debts?
- No clear methods of data collection, processing, and analysis. Please make sure the abstract clearly outlines these methods.
- You need to minimize the number of keywords to 5-6.

Introduction

- Page 1, Lines 2-3: "Bulgaria's demographics have undergone substantial shifts over the previous century." Please support this statement with a review of literature and include the statistical values of change or shifts from/to and the annual rate of change.
- Page 3, Lines 82-92: Although you have clearly articulated the three key contributions of your study, the objectives remain unclear. Why?
- Page 4 Lines 93-100: Delete

Materials and methods

- Page 4, Lines 102-130: They are completely part of your data processing, and I don't understand why you put them before the data acquisition. Please refine the structure of your data acquisition, processing, and analysis techniques. You are mixing things.
- Note: *** nothing is visible in all of your figures shown in the main body of the manuscript, not in the annex.
- This section needs major improvement, as the manuscript lacks clear descriptions of the scientific methods used for data acquisition, processing, analysis, and validation.

Results

- Page 12, Lines 338-367—Interdecadal variability of flows. I wonder if you could clearly depict the statistical variability and the triggering factors for the variability. The same is true for the regional and terrain migration dynamics section stated from lines 368-432.
- The results have not been validated. Please validate it.
- The quality of all your figures must be improved and saved into 300 dpi to enhance the visibility and ensure publication standards.
-

Discussion and Reflection

- Please include the findings when you discuss them. I didn't see any statistical values discussed in your manuscript.

Conclusion

- There is no explicit advice or recommendation regarding policy debt.
- The conclusion does not explicitly state the limitations of your study. Could you please include them?

Thank you.
